# A cohort study in Southern Xinjiang, China, 2018–2023 on the association of metabolic syndrome components and their interactions with cardiovascular disease risk

**DOI:** 10.3389/fcvm.2026.1727588

**Published:** 2026-03-05

**Authors:** Sijing Wang, Zumei Li, Xinyang Sun, Chu Cheng, Xiaofeng Han, Jingkai Mao, Dongqing An, Zhihao Zhang

**Affiliations:** 1 School of Traditional Chinese Medicine, Xinjiang Medical University, Urumqi, China; 2 Institute of Medical Engineering Interdisciplinary Research, Xinjiang Medical University, Urumqi, China; 3 School of Computer Science (National Pilot Software Engineering School), Beijing University of Posts and Telecommunications, Beijing, China; 4 The Fourth Affiliated Hospital of Xinjiang Medical University, Xinjiang Medical University, Urumqi, China; 5 School of Computer Science and Technology, Xinjiang University, Urumqi, China

**Keywords:** additive interaction, cardiovascular diseases, metabolic syndrome, retrospective cohort, survival analysis

## Abstract

The association between metabolic syndrome (MetS) and its components with cardiovascular disease (CVD) incidence represents a key global public health focus. However, given the unique dietary patterns of the population in southern Xinjiang, China, the differential effects of various Mets components on CVD onset and the interactions among these components remain poorly elucidated. This retrospective cohort study was conducted from 2018 to 2023, enrolling 144,966 participants from a county in southern Xinjiang with a median follow-up duration of 5.0 years. Cox proportional hazards regression and interaction analysis were applied to systematically explore the dose-response relationship between the number of abnormal values of the five core Mets indicators and CVD incidence. Among the 144,966 participants with no baseline CVD, the prevalence of Mets was 14.8%; Mets patients had a significantly higher mean age [(50.96 ± 13.28) vs. (40.30 ± 15.94) years, P<0.001]. During a median follow-up of 5.0 years, the incidence of CVD was 5.13% (51.3 cases/1,000 person-years) in the Mets group and 2.18% in the non-Mets group. After adjusting for confounders, elevated waist circumference (adjusted hazard ratio(aHR) = 1.12, 95%CI:1.08–1.15, P<0.001), elevated blood pressure (aHR = 1.24, 95%CI:1.20–1.29, P<0.001), and high blood glucose (aHR = 1.14, 95%CI:1.10–1.18, P<0.001) were independently associated with increased incident CVD events, while elevated triglycerides (aHR = 1.03, 95%CI:1.00–1.07, P=0.065) and low high-density lipoprotein cholesterol (aHR = 1.00, 95%CI:0.97–1.03, P=0.8788) showed no significant effect. Incident CVD events increased progressively with the number of abnormal Mets components (aHR = 1.22–2.72, P for trend <0.001). Significant positive additive interaction was observed between waist circumference and blood pressure, but not between other component pairs. These findings underscore the value of integrated waist circumference and blood pressure management for CVD prevention in similar populations, though recall bias and limited causal inference exist due to the retrospective design.

## 1 Introduction

Cardiovascular disease (CVD) is one of the leading causes of death and disability worldwide [1]. However, this disease is largely preventable, making the promotion of effective prevention and control a priority in public health practice [2]. metabolic syndrome (MetS) refers to a cluster of metabolic disorders including abdominal obesity, hypertension, hyperglycemia, dyslipidemia, and insulin resistance [4]. Future projections from 2022 to 2046 indicate that the burden of metabolic and cardiovascular diseases will continue to rise [3], with the increase in stroke-related burden alone expected to reach 55.9%. This highlights the imperative of targeted interventions [5]. Numerous studies have systematically demonstrated the close association between Mets and CVD [6], with the number of metabolic abnormalities showing a dose-response relationship with increased incident CVD events [7]. Patients with Mets have more than twice the risk of developing CVD compared to healthy individuals [8], and this excess risk is mainly attributed to the synergistic effects of various components of Mets. However, the specific contributions of each MetS component to CVD, as well as their interactions, vary across populations with distinct lifestyle and dietary [9] backgrounds—and these nuances have not been fully clarified.

Located in western China, Xinjiang is notable for a traditional dietary pattern dominated by high fat and salt intake alongside low dietary fiber content [10], a nutritional profile that drives elevated regional prevalence of metabolic syndrome (MetS), hypertension, type 2 diabetes mellitus, and dyslipidemia among indigenous populations [4, 11]. Despite the plausible link between this distinct dietary exposure and the onset of metabolic disturbances, systematic investigations into the underlying pathogenic mechanisms and the specific association between MetS and cardiovascular disease (CVD) in this population remain inadequately addressed. The present large-scale retrospective cohort study was therefore designed to fill these critical knowledge gaps by pursuing three core objectives: first, to delineate the associations between individual MetS components and incident CVD; second, to quantify the dose-response relationship between the number of abnormal MetS components and CVD risk; and third, to elucidate the additive interactions among these components.

## 2 Data and methods

### 2.1 Study design and population

**Figure 1:**
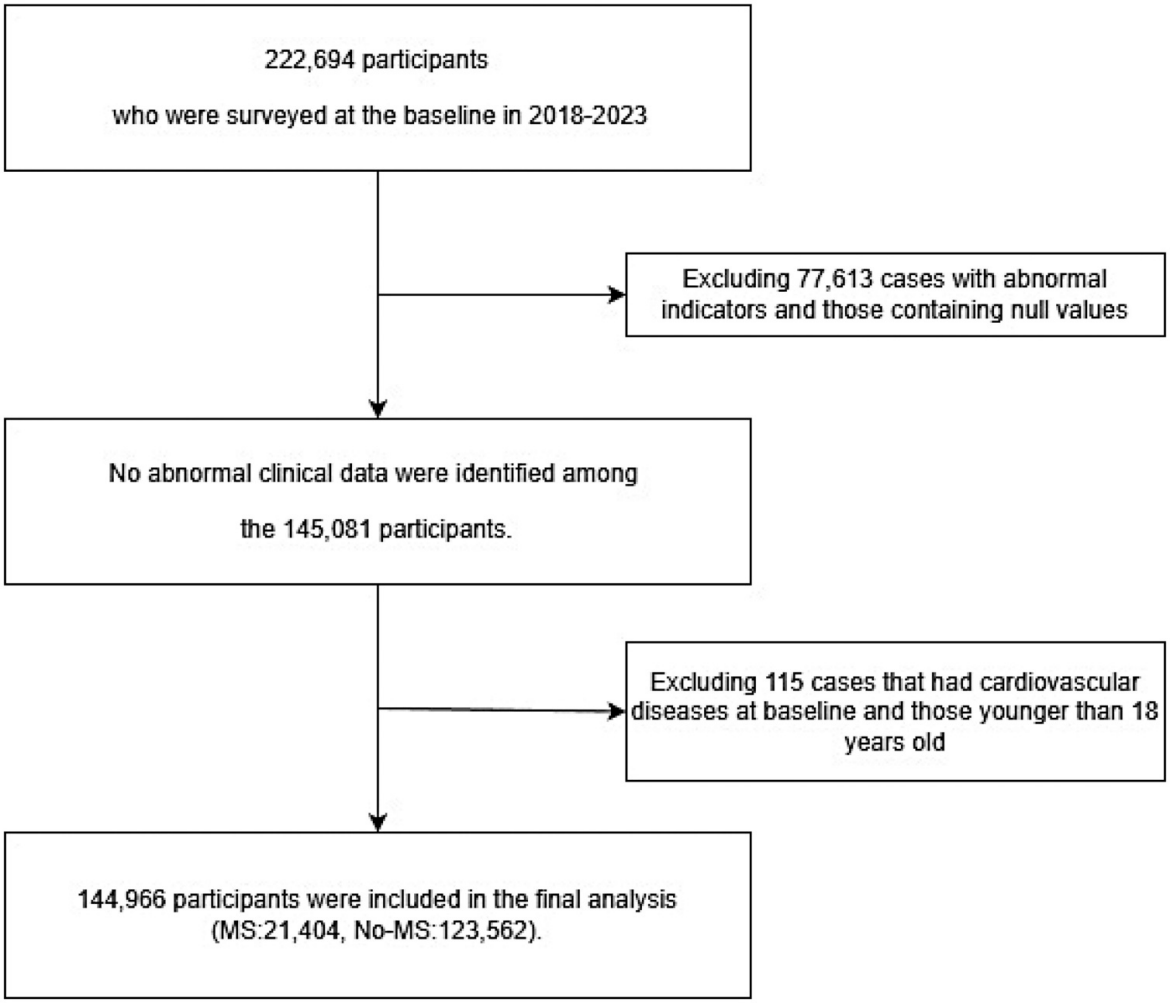
Flow chart of study participant screening. A total of 222,694 baseline participants (2018–2023) were included, excluding 77,613 with abnormal indicators and missing data, and 115 aged <18 years or with baseline cardiovascular diseases, finally enrolling 144,966 eligible participants.

We performed a large-scale retrospective observational cohort study using health check-up records obtained from a county hospital in southern Xinjiang, China, where data were collected continuously from 2018 to 2023. A retrospective design was adopted in this study, as it is more feasible than a prospective design, primarily due to the advantages of cost savings and convenient acquisition of a large sample size. The target population consisted of permanent residents aged ≥18 years in a county in Xinjiang. Individuals who were unwilling to cooperate, unconscious, suffering from other major diseases, or pregnant were excluded. In this study, participants without a prior diagnosis of CVD first confirmed between 2018 and 2020 were defined as the baseline population. According to the definition of the World Health Organization clinical guidelines, extreme abnormal values outside the physiologically reasonable range of each indicator are identified and excluded, and the specific ranges include: Systolic blood pressure >250 mmHg or <80 mmHg, diastolic blood pressure >150 mmHg or <50 mmHg, waist circumference >150 cm or <50 cm, triglyceride >10 mmol/L or <0.3 mmol/L, high density lipoprotein cholesterol <0.5 mmol/L or >3.0 mmol/L, blood glucose >20 mmol/L or <2.5 mmol/L. A total of 222,694 initial participants completed the baseline survey between 2018 and 2023; 77,613 participants with abnormal indicators or missing data were excluded, leaving 145,081 participants with complete and normal clinical data. Further exclusion of 115 individuals who had a confirmed diagnosis of CVD at baseline or were aged <18 years resulted in a final study cohort of 144,966 participants for the final analysis. The study design is illustrated in Figure 1.

### 2.2 Collection of baseline information and definition of confounders

The collected characteristic variables included demographic and behavioral indicators such as gender, survey year, age, exercise frequency, smoking status, dietary habits, and alcohol consumption frequency; anthropometric indicators including waist circumference (WC) and body mass index (BMI); biochemical indicators such as blood glucose (BG), total cholesterol (TC), triglycerides (TG), serum low-density lipoprotein cholestero(LDL-C), serum high-density lipoprotein cholestero(HDL-C), systolic blood pressure (SBP), and diastolic blood pressure (DBP); and past medical history indicatorsincluding history of diabetes mellitus and history of hypertension.

Traditional risk factors were used as confounders in the data analysis, specifically including age, gender, alcohol consumption frequency, exercise frequency, history of hypertension, history of diabetes mellitus, smoking status, dietary habits, and BMI.

### 2.3 Outcome assessment

Participants who experienced their first episode of ischemic or hemorrhagic stroke, coronary heart disease (CHD), or heart failure during the follow-up period were diagnosed with CVD. CHD was diagnosed if participants underwent coronary intervention (cardiac catheterization or coronary artery bypass grafting), developed angina pectoris, suffered a myocardial infarction, or were hospitalized for congestive heart failure during follow-up. Data on CVD events were obtained from hospital medical records (validated by ICD-10 codes: CHD I20-I25, stroke I60-I69, heart failure I50) and questionnaire interviews. If the same type of event occurred twice or more, the first occurrence was taken as the endpoint.

### 2.4 Definition of metabolic syndrome

According to the criteria established by the National Cholesterol Education Program Adult Treatment Panel III (NCEP-ATP III), Mets was defined as meeting at least three of the following five indicators: (1) abdominal obesity, defined as a waist circumference ≥102 cm (40 inches) in men and ≥88 cm (35 inches) in women; (2) elevated triglycerides, with a fasting serum triglyceride level ≥1.70 mmol/L (150 mg/dL); (3) reduced HDL-C, defined as <1.04 mmol/L (40 mg/dL) in men and <1.30 mmol/L (50 mg/dL) in women; (4) elevated blood pressure, with a systolic blood pressure ≥130 mmHg or diastolic blood pressure ≥85 mmHg, or a confirmed diagnosis of hypertension with ongoing antihypertensive treatment; (5) elevated fasting glucose, with a fasting blood glucose level ≥5.6 mmol/L (100 mg/dL), or a confirmed diagnosis of diabetes mellitus.

### 2.5 Statistic analysis

Continuous variables were presented as mean ± standard deviation (SD). Differences between groups were assessed using the independent samples t-test. Categorical variables were reported as frequencies and proportions. Differences between groups for categorical variables were evaluated using the chi-square test or Fisher’s exact test. Statistical significance was set at P<0.05. Sample size was estimated using the normal approximation method for cohort studies with binary outcomes, with a significance level (α) of 0.05, a statistical power of 0.90, and adjustment for the actual dropout rate. All statistical analyses were performed using Python 3.12.

Kaplan-Meier survival analysis was performed to estimate the CVD-free survival probabilities of participants with metabolic syndrome (Mets group) and without metabolic syndrome (No-Mets group), with the log-rank test used to compare the statistical significance of differences in survival curves between the two groups.

A Cox proportional hazards regression model was constructed to analyze the association between individual Mets components and incident CVD events, with CVD onset as the dependent variable and individual Mets components as the primary independent variables. Two analytical models were constructed: a crude model without adjustment for potential confounders and an adjusted model. The adjusted model incorporated confounding factors, including age, gender, smoking status, alcohol consumption frequency, exercise frequency, dietary habits, and body mass index (BMI), to control for their potential confounding effects on the association between Mets components and incident CVD events. Prior to model fitting, the proportional hazards assumption of the Cox model was verified using Schoenfeld residual tests, and no significant violations were observed.

The Cochran-Armitage trend test was applied to verify the linear trend between the number of Mets components and incident CVD events, thereby demonstrating the cumulative effect of the number of Mets components. Hazard ratio (HR) and adjusted hazard ratio (aHR) with 95% confidence intervals (CIs) from the Cox model were calculated to quantify the strength of associations, and the model was further extended to analyze the joint effects among high-risk Mets components. The relative excess risk of interaction (RERI), percentage attributable to interaction (AP) and synergy index (SI) were used to quantitatively analyze and describe the additive interaction effect between MetS components.

## 3 Results

### 3.1 Data loss/retention statistics

To evaluate the completeness of follow-up data, loss to follow-up was defined as the absence of subsequent physical examination records from the time of first enrollment to the end of the study period. A total of 144,966 eligible participants were included in the final baseline cohort.

Overall, 14,124 participants were lost to follow-up, corresponding to an overall loss rate of 9.74% and a retention rate of 90.26% (Table 1). This indicated a high completeness of follow-up data for the study cohort. Stratified by the year of first examination, the loss to follow-up rate exhibited a gradual upward trend with later enrollment. Participants first examined in 2023 had a 100% loss rate, as no follow-up data were available for this cohort by the end of the study period.

**Table 1:** Overall and stratified loss/retention statistics by year of first examination.

First exam year	Total (*n*)	Lost (*n*)	Loss rate (%)	Retained (*n*)	Retention rate (%)
2018	90,641	2,755	3.04	87,886	96.96
2019	32,548	2,599	7.99	29,949	92.01
2020	9,216	2,104	22.83	7,112	77.17
2021	3,941	1,032	26.19	2,909	73.81
2022	3,820	834	21.83	2,986	78.17
2023	9,597	9,597	100.00	0	0.00
**Overall**	**144,966**	**14,124**	**9.74**	**130,842**	**90.26**

The bolded text represents the summary results.

### 3.2 Baseline characteristics comparison

In this study, the Mets group comprised 21,404 participants, while the non-Mets group included 123,562. As presented in Table 2, statistically significant differences were observed in all baseline characteristics between the two groups (all P-values < 0.001).

**Table 2:** Baseline characteristics of study participants.

Variable	Mets (n = 21,404)	Non-Mets (n = 123,562)	P-value
Age (years)	50.96±13.28	40.30±15.94	<0.001
BMI	27.47±4.62	23.56±3.90	<0.001
Gender (%)			<0.001
Male	8250(38.5)	60968(49.3)	
Female	13154(61.5)	62594(50.7)	
Exercise frequency (%)			<0.001
No exercise	20338(95.0)	115773(93.7)	
Daily	662(3.1)	4953(4.0)	
More than once a week	65(0.3)	305(0.2)	
Occasionally	339(1.6)	2531(2.0)	
Smoking status (%)			<0.001
Never smoked	18967(88.6)	105442(85.3)	
Current smoker	2203(10.3)	16875(13.7)	
Former smoker	234(1.1)	1245(1.0)	
Dietary habits (%)			<0.001
Balanced diet	16512(77.1)	94076(76.1)	
Vegetarian-dominant	3722(17.4)	22555(18.3)	
Others	200(0.9)	1611(1.3)	
Meat-dominant	970(4.5)	5320(4.3)	
Alcohol consumption frequency (%)			<0.001
Never	19533(91.3)	110902(89.8)	
Occasionally	1711(8.0)	11288(9.1)	
Frequently	143(0.7)	1181(1.0)	
Daily	17(0.1)	191(0.2)	
History of hypertension (%)			<0.001
No	20004(93.5)	122420(99.1)	
Yes	1400(6.5)	1142(0.9)	
History of diabetes mellitus (%)			<0.001
No	21117(98.7)	123426(99.9)	
Yes	287(1.3)	136(0.1)	

Compared with the non-Mets group, the Mets group was characterized by older age and higher BMI. In terms of demographic and lifestyle factors, the Mets group had a higher proportion of females, lower levels of physical activity, and lower smoking and alcohol consumption rates. Notably, the prevalence of chronic diseases was substantially higher in the Mets group, with markedly elevated proportions of hypertension (6.5% vs. 0.9%) and diabetes mellitus (1.3% vs. 0.1%) compared to the non-Mets group.

### 3.3 CVD cumulative incidence and CVD-free survival analysis by mets status

Over a median follow-up of 5.0 years, the cumulative incidence of cardiovascular disease (CVD) in the Mets group was significantly higher than that in the No-Mets group (5.13% vs. 2.18%, P<0.001). The Mets group had a cumulative incidence of 51.3 per 1,000 participants, and also exhibited a steeper decline in CVD-free survival probability. Based on these incidence rates, a relative risk (RR) of 2.35, a sample size ratio of 5.8:1 between the No-Mets and Mets groups, along with a significance level of α=0.05, a statistical power of 0.90, and an actual dropout rate of 9.74%, the estimated minimum total sample size was 4,747 cases. However, our study enrolled 144,966 independent individuals (30.54 times the required sample size), ensuring sufficient statistical power to detect the target associations.

Over a median follow-up of 5.0 years, the cumulative CVD incidence in the Mets group was significantly higher than that in the No-Mets group (5.13% vs. 2.18%, P<0.001), corresponding to an incidence density of 51.3 cases per 1,000 person-years in the MetS group. Additionally, the MetS group demonstrated a more rapid decline in CVD-free survival probability.

**Figure 2:**
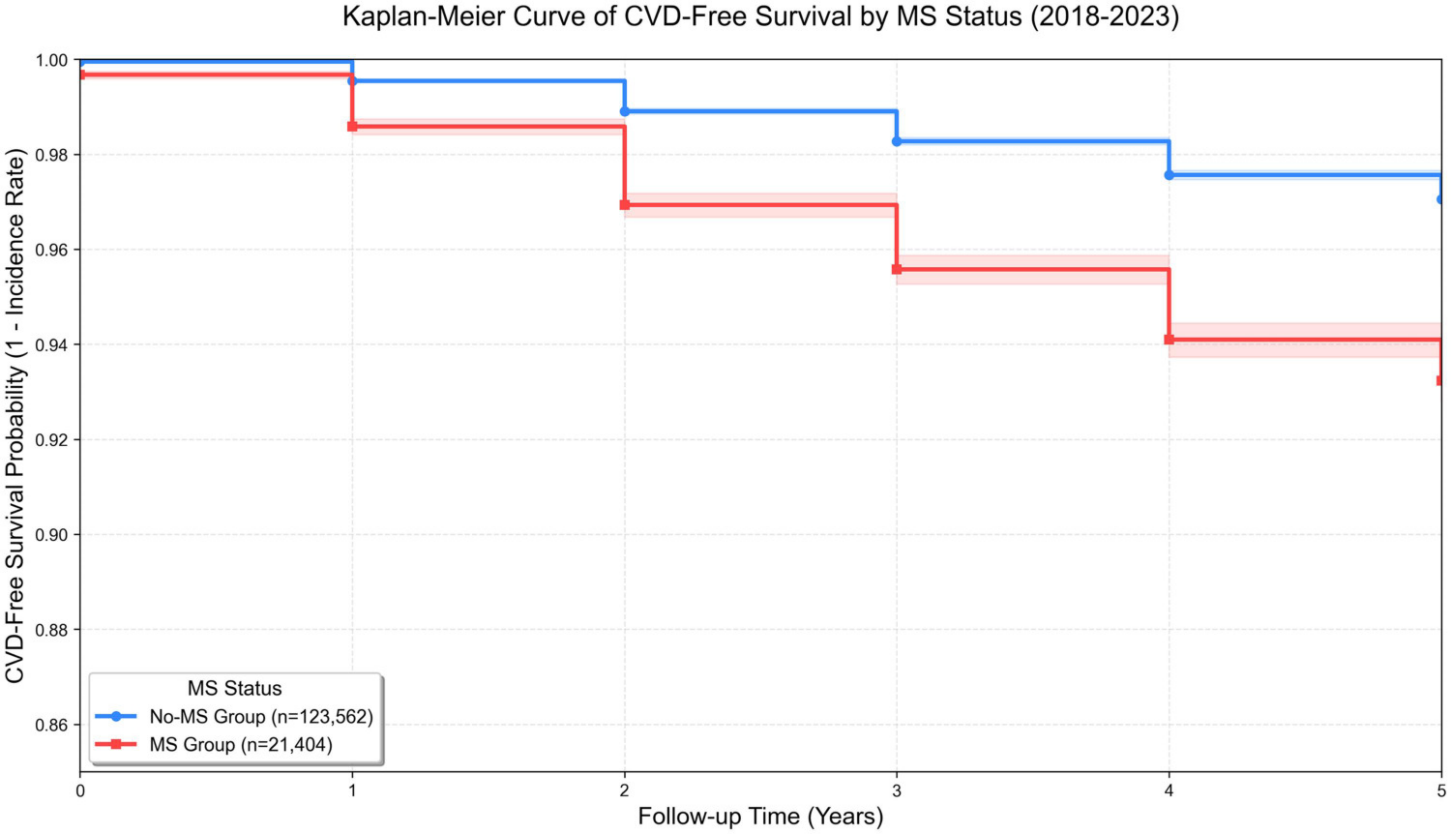
Kaplan-Meier curves of CVD-free survival by Mets status.

Kaplan-Meier survival analysis was performed to compare CVD-free survival between participants with metabolic syndrome (Mets group, *n* = 21,404, 14.76%) and without metabolic syndrome (No-Mets group, *n* = 123,562, 85.24%), as shown in Figure 2. The log-rank test revealed a statistically significant difference in survival curves between the two groups ($\chi^{2}=656.69$, P<0.001). Table 3 demonstrates that annual CVD incidence rates were consistently higher in the Mets group throughout the follow-up period.

**Table 3:** Annual at-risk population and CVD incidence by Mets status (5-Year follow-up).

Follow-up	No-Mets	No-Mets	Mets	Mets
Year	At-risk (*n*)	CVD incidence (%)	At-risk (*n*)	CVD incidence (%)
1	123,562	0.05	21,404	0.33
2	111,574	0.41	19,148	1.09
3	102,729	0.64	17,320	1.68
4	94,676	0.64	15,872	1.40
5	83,674	0.72	13,980	1.55

No-Mets, without metabolic syndrome; Mets, with metabolic syndrome; At-risk (*n*), number of participants under follow-up at each time point; CVD Incidence (%), annual CVD incidence rate.

### 3.4 The association between each component of Mets and CVD

Table 4 presents the results of the dynamic cohort analysis regarding the association between individual components of metabolic syndrome and incident CVD events: in the unadjusted model, elevated BP, elevated WC, elevated TG, and elevated BG were all significantly associated with incident CVD events, with unadjusted HR of 2.73, 1.81, 1.2, and 1.85, respectively (all corresponding P-values < 0.001). However, reduced HDL-C was not significantly associated with incident CVD events, with a corresponding P-value of 0.9378.

**Table 4:** Association between Mets components and incident CVD events.

Mets components	(HR (95%CI)	P-value (unadj)	aHR (95%CI)	P-value (adj)
Elevated BP	2.73 (2.56–2.91)	<0.001	1.24 (1.20–1.29)	<0.001
Elevated WC	1.81 (1.70–1.94)	<0.001	1.12 (1.08–1.15)	<0.001
Elevated TG	1.20 (1.12–1.29)	<0.001	1.03 (1.00–1.07)	0.0651
Reduced HDL-C	1.00 (0.94–1.07)	0.9378	1.00 (0.97–1.03)	0.8788
Elevated BG	1.85 (1.73–1.98)	<0.001	1.14 (1.10–1.18)	<0.001

After adjustment for confounders, elevated BP, elevated WC, and elevated BG remained significantly associated with incident CVD events, with aHR of 1.24, 1.12, and 1.14, respectively (all corresponding P-values < 0.001). Nevertheless, the association of elevated TG with incident CVD events was no longer statistically significant (corresponding P-value = 0.0651), and the association between reduced HDL-C and incident CVD events also showed no significance (corresponding P-value = 0.8788).

### 3.5 The association between the quantities of each component of Mets and CVD

Table 5 analyzed the dose-response association between the number of Mets components and the incident CVD event. Taking individuals without any Mets components as the reference group, the incidence rate of CVD was 1.4% with the aHR set at 1.00. As the number of Mets components increased, the incidence rate of CVD showed a graded elevation: 1.9% for 1 component, 2.7% for 2 components, 4.1% for 3 components, 6.2% for 4 components, and 6.9% for 5 components. The corresponding adjusted hazard ratios increased synchronously, which were 1.22, 1.49, 1.82, 2.23 and 2.72 for 1, 2, 3, 4 and 5 components respectively. The HR also showed an increasing trend with the increase in the number of components. The results of the trend test showed that P<0.001, confirming that there was a statistically significant dose-response relationship between the number of Mets components and the incident CVD event, i.e., the more Mets components an individual had, the higher the risk of developing CVD.

**Table 5:** Association between number of Mets components and incident CVD events.

Mets components group	Total	CVD cases	Incidence (%)	HR (95%CI)	aHR (95%CI)
0	25,020	343	1.4	Reference	Reference
1	49,842	971	1.9	1.36 (1.20–1.54)	1.22 (1.19–1.26)
2	40,550	1,090	2.7	1.93 (1.71–2.18)	1.49 (1.41–1.58)
3	21,071	854	4.1	2.93 (2.58–3.32)	1.82 (1.68–1.98)
4	7,173	442	6.2	4.43 (3.85–5.10)	2.23 (2.00–2.48)
5	1,310	90	6.9	4.93 (3.91–6.22)	2.72 (2.38–3.12)

Grouping is based on the number of abnormal core components of Mets (WC, BP, BG, TG, HDL-C), with Group 0 having no abnormal components and Groups 1-5 having 1-5 abnormal components respectively. *P* for trend <0.001 in both unadjusted and adjusted models.

### 3.6 Analysis of the interaction between high-risk components and CVD

Table 6 analyzed the association between different combined exposure patterns of core Mets components (BG, WC, and BP) and incident CVD events, as TG and HDL-C were not statistically significant after adjustment and thus not included in the core combined exposure analysis. With the combination of normal levels of all components [BG(−) & WC(−) & BP(−)] as the reference group, the results showed that the CVD incidence rates per 100 person-years and adjusted hazard ratios (aHRs) for the BG-WC, BG-BP, and WC-BP combinations all showed a graded increase with the presence of combined exposure. Specifically, the incidence rates per 100 person-years in the “BG(+) & WC(+),” “BG(+) & BP(+),” and “WC(+) & BP(+)” groups reached 0.2872, 0.4409, and 0.4200, respectively, with corresponding aHRs of 2.10, 2.74, and 3.31. All these aHRs were higher than the risk levels of the single-exposure subgroups within each combination, suggesting that the incident CVD events associated with combined exposure to abnormal Mets components is higher than that of exposure to individual components.

**Table 6:** Association between combined exposures to abnormal Mets components and incident CVD events.

Exposure category	Event rate per 100 PY	HR (95% CI)	aHR (95% CI)
BG(−) & WC(−)	0.0893	Reference	Reference
BG(−) & WC(+)	0.1803	2.02 (1.86–2.19)	1.74 (1.60–1.89)
BG(+) & WC(−)	0.1812	2.03 (1.82–2.26)	1.30 (1.17–1.45)
BG(+) & WC(+)	0.2872	3.22 (2.95–3.51)	2.10 (1.93–2.30)
BG(−) & BP(−)	0.0728	Reference	Reference
BG(−) & BP(+)	0.3183	4.38 (4.04–4.73)	2.35 (2.16–2.55)
BG(+) & BP(−)	0.1253	1.72 (1.56–1.91)	1.34 (1.21–1.49)
BG(+) & BP(+)	0.4409	6.06 (5.58–6.58)	2.74 (2.51–3.00)
WC(−) & BP(−)	0.0634	Reference	Reference
WC(−) & BP(+)	0.2711	4.28 (3.85–4.75)	2.00 (1.79–2.23)
WC(+) & BP(−)	0.1089	1.72 (1.55–1.90)	1.50 (1.35–1.66)
WC(+) & BP(+)	0.4200	6.62 (6.05–7.25)	3.31 (3.01–3.64)

(+), elevated/high level; (–), normal/low level; Event Rate per 100 PY: CVD incidence rates per 100 person-years.

Table 7 further evaluated the additive interactions among Mets components. The combinations of BG & waist WC and BG & BP showed RERI of 0.0654 and 0.0557, respectively, with 95% confidence intervals (CIs) crossing 0. Their AP were 0.0311 and 0.0203, respectively, and the corresponding 95% CIs also spanned 0. Additionally, the SI were 1.0630 and 1.0330 (both close to 1), indicating no statistically significant additive interaction for these two pairs. In contrast, the WC & BP combination exhibited a significant positive additive interaction (synergistic effect), with an RERI of 0.8136 (95% CI: 0.6366–0.9907), an AP of 0.2456 (95% CI: 0.1917–0.2995), and an SI of 1.5427 (95% CI: 1.3763–1.7092). These results suggest that the effect of combined exposure to abnormal WC and BP on incident CVD events exceeded the sum of their individual effects, and this synergistic effect was statistically significant.

**Table 7:** Additive interaction measures of Mets components on incident CVD events (adjusted model).

Interaction	RERI (95% CI)	AP (95% CI)	SI (95% CI)
BG & WC	0.0654 (−0.0974–0.2282)	0.0311 (−0.0463–0.1085)	1.0630 (0.8992–1.2267)
BG & BP	0.0557 (−0.1042–0.2157)	0.0203 (−0.0380–0.0787)	1.0330 (0.9361–1.1300)
WC & BP	0.8136 (0.6366–0.9907)	0.2456 (0.1917–0.2995)	1.5427 (1.3763–1.7092)

### 3.7 Sensitivity analysis

To evaluate the potential impact of loss to follow-up on the robustness of core findings, a sensitivity analysis was performed by excluding all 14,124 participants lost to follow-up, based on a validated dataset comprising 130,842 unique individuals with statistical methods consistent with the primary analysis. In this sensitivity analysis cohort, 111,629 participants were metabolic syndrome-free (${\mathrm{MetS}}^{-}$), with 2,689 incident CVD cases (incidence rate: 2.41%), while 19,213 participants were diagnosed with metabolic syndrome (${\mathrm{MetS}}^{+}$), with 1,093 incident CVD cases (incidence rate: 5.69%). Kaplan-Meier survival analysis with log-rank test confirmed that the difference in CVD-free survival curves between the two groups remained statistically significant (P<0.001).

Repeated Cox proportional hazards regression analyses showed that after adjusting for confounding factors, elevated blood pressure (aHR = 1.26, 95%CI: 1.22–1.31, P<0.001), elevated waist circumference (aHR = 1.12, 95% CI: 1.09–1.16, P<0.001), and elevated blood glucose (aHR = 1.15, 95%CI: 1.11–1.19, P<0.001) remained independent risk factors for CVD, whereas elevated triglycerides (aHR = 1.03, 95%CI: 1.00–1.07, P=0.06) and reduced high-density lipoprotein cholesterol (aHR = 1.00, 95%CI: 0.97–1.03, P=0.8624) showed no statistically significant association with CVD risk.

A significant dose-response relationship between the number of abnormal MetS components and CVD incidence was still observed (*P* for trend <0.001); with participants having 0 abnormal components as the reference (aHR = 1.00), the adjusted hazard ratios for those with 1, 2, 3, 4, and 5 abnormal components were 1.22 (95%CI: 1.19–1.25), 1.49 (95%CI: 1.41–1.57), 1.82 (95%CI: 1.68–1.97), 2.22 (95%CI: 1.99–2.47), and 2.71 (95%CI: 2.37–3.1), respectively, with the magnitude of aHR values almost identical to that in the primary analysis (variation range <1%). In addition, the positive additive interaction between elevated waist circumference and elevated blood pressure remained statistically significant, with a relative excess risk due to interaction (RERI) of 0.8075 (95%CI: 0.6304–0.9847), an attributable proportion due to interaction (AP) of 0.2443, and a synergy index (SI) of 1.5392, which were highly consistent with the primary analysis results.

Overall, the association strength between MetS (and its core components including elevated waist circumference, elevated blood pressure, and elevated blood glucose) and CVD risk, the dose-response relationship between the number of MetS components and CVD incidence, and the positive additive interaction between elevated waist circumference and elevated blood pressure showed no substantial deviation from the primary analysis results. The variation amplitude of core adjusted hazard ratios was less than 1% with highly overlapping 95% confidence intervals, indicating that loss to follow-up did not exert a material impact on the core conclusions of this study, thus verifying the good robustness and reliability of the findings.

## 4 Discussion

Based on 5-year median follow-up data of 144,966 adult residents from a county in southern Xinjiang, China, this study systematically analyzed the associations between Mets and its components with the incident CVD event. It identified the key Mets components associated with CVD, as well as the characteristics of their cumulative effects and interactions. The strengths of this study include a large sample size, long-term follow-up, systematic analysis of interactions among Mets components, and the use of data derived from physical examination records which ensures high objectivity. These findings provide empirical evidence for the precision prevention and control of CVD in this region.

### 4.1 The relationship between Mets and its components and CVD

This study found that the incidence of CVD in the Mets group was significantly higher than that in the non-Mets group (5.13% vs. 2.18%, P<0.001), which is consistent with numerous previous studies [6] and further validates the public health significance of Mets as an aggregated marker of incident CVD events.

At the level of Mets components, elevated BP, elevated WC [12], elevated TG [13], and elevated BG all independently increase incident CVD events. Specifically, after adjusting for confounding factors, the association between elevated triglycerides and incident CVD events was no longer statistically significant. Among these components, the association between WC and incident CVD events was particularly prominent after adjustment, indicating that elevated WC may be a key predictor of incident CVD events in the study population of southern Xinjiang. This finding is consistent with domestic epidemiological studies, which have indicated that central obesity are closely associated with an increased risk of CVD than BMI [14, 15]. In terms of pathological mechanisms, the pathogenic mechanisms of individual Mets components are characterized by multi-target damage: elevated BP impairs vascular endothelium and increases the risk of atherosclerosis [16], while elevated BG may inhibit myocardial contraction [17].

This study did not observe a significant independent association between decreased HDL-Cand incident CVD events. The discrepancy between this finding and traditional knowledge may be because the degree of HDL-C reduction did not reach the risk threshold, and its protective effect was masked by the synergistic interactions of other components in Mets. Some studies have also pointed out that HDL-C has high functional complexity, and its cardiovascular protective effect may not be determined solely by concentration but also related to factors such as HDL-C particle size and functional status [19]. Notably, a recent study further expanded this perspective: instead of focusing on a single HDL-C index (which is susceptible to confounding factors and cannot accurately predict CVD prognosis independently), it identified the hs-CRP/HDL-C ratio as a significant risk factor for CVD [18]. Their findings suggest that future research could incorporate this inflammation-lipid composite marker, in addition to combining metabolomics techniques, to further analyze the dose-response relationship between HDL-C functional phenotypes and incident CVD events.

### 4.2 The dose-response relationship between the quantity of metabolic syndrome components and the incident CVD event

This study demonstrated that the incident CVD events increases with the number of Mets components. This result is highly consistent with the theory of cumulative pathogenicity of Mets components proposed by Ford ES [20]. From a biological mechanism perspective, an increase in the number of Mets components essentially reflects the “synergistic amplification effect” of multiple metabolic disorders [8, 21].

The dose-response relationship at the epidemiological level has important guiding value for clinical practice: it suggests that Mets is a holistic exposure factor for incident CVD events, and the number of components should be comprehensively evaluated rather than focusing on a single indicator in isolation. For Mets patients with multiple high-risk components, precision strategies combining intensified lifestyle interventions and pharmacotherapy should be adopted to interrupt the chain of synergistic damage caused by metabolic disorders.

### 4.3 The additive interaction among components of metabolic syndrome

The additive interaction analysis of this study confirmed that synergistic pathogenic effects exist between every pair of BP, waist WC, and BG, with the interaction between BP and WC being the most significant. This finding challenges the traditional understanding of pathogenesis driven by individual components and provides new targets for the “precision stratified management” of incident CVD events.

From a biological mechanism perspective, the aforementioned synergistic effects have a reasonable pathophysiological basis. First, the interaction between BP and WC may arise from the interplay and superimposition of increased vascular wall pressure caused by hypertension and chronic inflammation and metabolic disorders induced by central obesity. These factors jointly impair vascular regulation and repair capabilities, thereby significantly accelerating the progression of atherosclerosis [22]. Second, the interaction between WC and BG is mainly attributed to lipid metabolism disorders caused by central obesity, which coexist with insulin resistance. Together, they lead to an imbalance in glucose synthesis and clearance, exacerbating cardiovascular damage [23]. Additionally, the interaction between BP and BG can be explained as follows: vascular endothelial dysfunction caused by hypertension impairs the normal transport of lipids, while elevated BG further damages the cardiovascular system by increasing blood viscosity and inflammatory responses [6].

These findings indicate that when assessing incident CVD events, e should go beyond the limitations of individual indicators and comprehensively consider the combined effects of multiple metabolic abnormalities. This provides new insights for the precise risk assessment and stratified management of CVD.

### 4.4 The limitations of the research and its future directions

Although this study, based on large-scale retrospective cohort data of 144,966 adult residents, revealed significant associations between Mets and its components with incident CVD events, it still has several limitations. First, the study data were mainly derived from a specific population (residents of a county in southern Xinjiang, China), which may introduce selection bias; therefore, the generalizability of the results should be interpreted with caution. Second, some variables (e.g., dietary habits and exercise frequency) were collected via self-report, which may lead to recall bias. Additionally, as a retrospective study, it cannot fully control all confounding factors nor establish a causal relationship between Mets and CVD.

Future studies should adopt a prospective cohort design to verify the generalizability of the current findings in Xinjiang populations and explore region-specific associations. Furthermore, integrating multi-omics approaches (e.g., metabolomics and genomics) may help unravel the deeper mechanistic links between Mets and CVD [24]. Meanwhile, based on the characteristics of interactions between Mets components and CVD in Xinjiang populations, combining epidemiological evidence with clinical practice to develop region-specific guidelines for the early screening of CVD in southern Xinjiang is a worthwhile endeavor.

## 5 Conclusion

This large-scale retrospective cohort study confirmed that elevated waist circumference, blood pressure, and blood glucose are independent risk factors for incident CVD in southern Xinjiang populations with a high fat/salt dietary pattern, while elevated triglycerides and reduced HDL-C showed no significant associations with CVD risk. A clear dose-response relationship was observed between the number of abnormal MetS components and CVD incidence, with CVD risk increasing progressively as the number of abnormal components rose. Most importantly, a significant positive additive interaction was identified between elevated waist circumference and blood pressure, indicating that their combined effect on CVD risk exceeds the sum of individual effects.

Based on these findings, integrated management of waist circumference and blood pressure should be prioritized in CVD prevention strategies for this population: (1) Targeted lifestyle interventions: Develop region-specific dietary guidelines to reduce high fat/salt intake (e.g., limiting mutton fat and pickled food consumption) and promote moderate physical activity (e.g., 150 min of weekly moderate-intensity exercise) to control abdominal obesity; (2) Clinical screening and early intervention: Incorporate simultaneous assessment of waist circumference and blood pressure into routine health check-ups, and implement combined pharmacological and non-pharmacological interventions for individuals with both elevated waist circumference and hypertension; (3) Community-based prevention programs: Establish community health management systems to monitor waist circumference and blood pressure in high-risk groups (e.g., middle-aged and elderly populations with MetS) and provide personalized lifestyle guidance.

These targeted measures can effectively mitigate the synergistic CVD risk from abdominal obesity and hypertension, and provide evidence for CVD prevention in other populations with similar dietary and metabolic characteristics.

## Data Availability

The original contributions presented in the study are included in the article/Supplementary Material, further inquiries can be directed to the corresponding authors.
